# P04.27. Panax ginseng in randomized controlled trials: a systematic review

**DOI:** 10.1186/1472-6882-12-S1-P297

**Published:** 2012-06-12

**Authors:** J Shergis, T Zhang, W Zhou, C Xue

**Affiliations:** 1 Royal Melbourne Institute of Technology, Melbourne, Australia

## Purpose

Panax ginseng is a commonly used herb for improving stamina and vitality. Contemporary clinical studies have purported its usefulness for many conditions including physical and cognitive performance, cardiovascular risk factors and respiratory conditions. With several high quality preparations on the market it is timely to explore their effectiveness in clinical studies.

## Methods

A systematic review was conducted to identify randomised controlled trials evaluating mono-preparations of Panax ginseng compared to control in patients with any type of disease or in healthy individuals. Major databases (five in English and three in Chinese) were searched, as well as hand searches to identify suitable studies with no publication date restriction. Included studies were independently assessed by two reviewers using the Cochrane risk of bias tool.

## Results

Of the 414 potentially relevant studies, 67 met the inclusion criteria and were reviewed. The conditions investigated included: psychomotor performance (n=15), physical performance (n=10), circulatory system (n=9), diabetes and glucose metabolism (n=7), quality of life/ mood (n=6), respiratory system (n=5), immunology (n=4), erectile dysfunction (n=4), menopausal symptoms (n=2), anti-oxidant function (n=2), cancer (n=2) and dry mouth (n=1). Panax ginseng was compared to placebo in 62 studies, to conventional treatment in four studies and to no intervention in one study. The risk of bias was unclear in the majority of studies. A high level of heterogeneity was seen between studies, most notably differing preparations of Panax ginseng, therefore hindering pooling of results and confounding interpretation. Safety outcomes were evaluated in some studies with minor adverse events reported in 25 studies.

## Conclusion

The effectiveness of Panax ginseng varied for different conditions with no solid conclusions deduced. Some promising results can be seen for respiratory diseases and psychomotor performance. Due to Panax ginseng’s popularity further studies with larger sample sizes and stronger methodological quality are warranted to produce conclusive results.

